# Basal and LPS-stimulated inflammatory markers and the course of individual symptoms of depression and anxiety

**DOI:** 10.1192/j.eurpsy.2022.666

**Published:** 2022-09-01

**Authors:** W. Van Eeden

**Affiliations:** Leiden university medical center, Psychiatry, Leiden, Netherlands

**Keywords:** Anxiety, inflammation, Course trajectory, Depression

## Abstract

**Introduction:**

A cross-sectional relationship between low-grade inflammation –characterized by increased blood levels of C-reactive protein (CRP) and pro-inflammatory cytokines– and both MDD and anxiety has been reported, but the potential longitudinal symptom-specific relationships has been less well studied.

**Objectives:**

We aimed to test our hypothesize that inflammation is predictive of the severity and the course of a subset of MDD and anxiety symptoms, especially symptoms that overlap with sickness behavior, such as anhedonia, anorexia, low concentration, low energy, loss of libido, psychomotor slowness, irritability, and malaise.

**Methods:**

We tested the association between basal and lipopolysaccharide (LPS)-induced inflammatory markers with individual MDD symptoms (measured using the Inventory of Depressive Symptomatology Self-Report) and several measures for symptom domains of anxiety over a period of up to 9 years using multivariate-adjusted mixed models in up to 2872 Netherlands Study of Depression and Anxiety (NESDA) participants.

**Results:**

At baseline, 53.9% of the participants had a current mood or anxiety disorder. We found that basal and LPS-stimulated inflammatory markers were more strongly associated with sickness behavior symptoms at up to 9-year follow up compared to non-sickness behavior symptoms of depression. The associations with anxiety symptoms attenuated by 25%-30% after adjusting for the presence of (comorbid) MDD.

**Conclusions:**

Inflammation was related to the presence and the course of specific MDD symptoms, of which the majority overlaped with sickness behavior. The associations between inflammatory markers and anxiety symptoms were partly driven by co-morbid MDD. Anti-inflammatory strategies should be tested in the subgroup of MDD patients who report depressive symptoms related to sickness behavior.

**Disclosure:**

No significant relationships.

